# Association of behavior pattern with overweight and obesity in South Korean adults–A multi correspondence analysis (KNHANES-2018–2020)

**DOI:** 10.1371/journal.pgph.0002384

**Published:** 2023-09-18

**Authors:** Do Hee Kim, Vasuki Rajaguru, Bomgyeol Kim, Suk-Yong Jang, Jaeyong Shin, Sang Gyu Lee, Tae Hyun Kim

**Affiliations:** 1 Department of Public Health, Graduate School, Yonsei University, Seoul, South Korea; 2 Department of Healthcare Management, Graduate School of Public Health, Yonsei University, Seoul, South Korea; 3 Department of Preventive medicine, College of Medicine, Yonsei University, Seoul, South Korea; PLOS: Public Library of Science, UNITED STATES

## Abstract

The objective was to determine the association between health-related behaviour with overweight and obesity in South Korean adults by using the Korean National Health and Nutritional Examination Survey (KNHANES) 2018–2020. The study participants were 16,784 aged ≥ 20years. The variables were socio-demographic, lifestyle, food habits and metabolic conditions. The logistic regression analysis performed to find the association by the odds ratio (OR, 95% CI). MCA performed to identify risk factors were computed for overweight and obesity. Overweight and obesity were significantly associated with health behaviour, high income (OR = 1.26; 95% CI: 1.15–1.39), smoking(OR = 1.29; 95% CI: 1.08–1.53), low physical activity(OR = 3.23; 95% CI: 1.79–4.69), diabetes(OR = 2.70; 95% CI: 1.62–4.50), high cholesterol and low HDL(OR = 3.98; 95%CI:2.65–5.97). The high discriminant variables of MCA were aged over 60years, lower education, high income, diabetes, lack of physical activity, and high cholesterol. The findings confirm that the OR of obesity and overweight was likely associated with health behaviour patterns. Besides, it indicates the MCA would be very effective to identify the population-based data context than individual data and it may suggest that more research on association between health behaviours and obesity prevention interventions should be developed for each age group for better health outcomes.

## Introduction

Obesity is a global epidemic and public health problem affecting all age groups and the World Health Organization (WHO) states that worldwide obesity has almost tripled since 1975 [1]. It seems that 1 billion people are obese, amounting to about 650 million adults. It is estimated to increase approximately by 167 million by 2025 [1]. The prevalence of obesity has been increased rapidly over 10 years between 2009 and 2018 in the total South Korean population; 29.7% in 2009 and 35.7% in 2018, respectively across all age groups, especially in the 20s, and 80s or older [2].

Obesity is determined by body mass index (BMI), which is the most widely used method among indices using body weight and height because it correlates highly with body fat mass in the majority of the population. The WHO defined overweight as a BMI of 25kg/m^2^ or more and obesity as a BMI of 30kg/m2 or more, regardless of race or gender and obese adults have increased chances of multi-morbidity and mortality [3–5]. Thus, obesity can be a life-threatening condition. Previous studies have reported the association of obesity status with higher health expenditures or greater use of health services, accounting for 5.8% of the total cost [6–8].

The impact of overweight and obesity has been attributed to a complex mixture of biological, behavioural and environmental factors [9–14] and unfavourable health behaviours [15]. The biological factors include age, gender, and genetic aspects; behavioural factors include individual lifestyle, attitude, knowledge, and environmental factors. Environmental factors include income, health habits, dietary habits, exercise, smoking and alcohol intake etc [11]. And dietary patterns are associated with obesity [12].

The Korean Society for the Study of Obesity (KSSO) has devoted time and resources to investigating recent trends in obesity [16]. Most of the population-based studies have been analysed by a variety of statistical methods by regression models to identify between or within groups, individual behavioural characteristics, and age-standardized and regional-based prevalence that are most likely to be associated with overweight/obesity or chronic diseases. To the best of our knowledge, few studies have forecasted the future prevalence of obesity in South Korean adults using different statistical models, including factors contributing to the disease and related costs [2,5–7,17–20].

This study focused on a multi correspondence analysis (MCA) [21]. The MCA is a multivariate method, which distributes frequency values of a table in an N-dimensional space to establish the similarity of the degree of variables by using the distance between the variables in each dimension [22]. MCA is free from assumptions and, working with categorical data, it may represent linear and non-linear relationships equally well [21]. MCA is not very common in public health articles in South Korea. It has never been used to analyse overweight or obesity. As far as we know, it has been used to analyse population-based data well to find the association, and it can also be consulted in different studies in the healthcare delivery system.

Despite it being a public health concern, this study built on the previous literature and investigated the association between obesity and behaviour pattern outcomes among people in pacific island [23] who are having remarkable behaviour pattern and focused whether health behaviour pattern moderated the association with body weight outcomes. This is an attempt to explain how the results have been inconsistent when analysing health behaviour pattern and obesity according to the standard classifications of BMI by MCA method. This cross-sectional study aims to find the association between behaviour pattern and obesity by MCA in a standardized population-based sample of South Korean adults using data from the Korea National Health and Nutrition Examination Survey (KNHANES) from 2018 to 2020.

## Methods

### Data source and population

**Fig 1:**
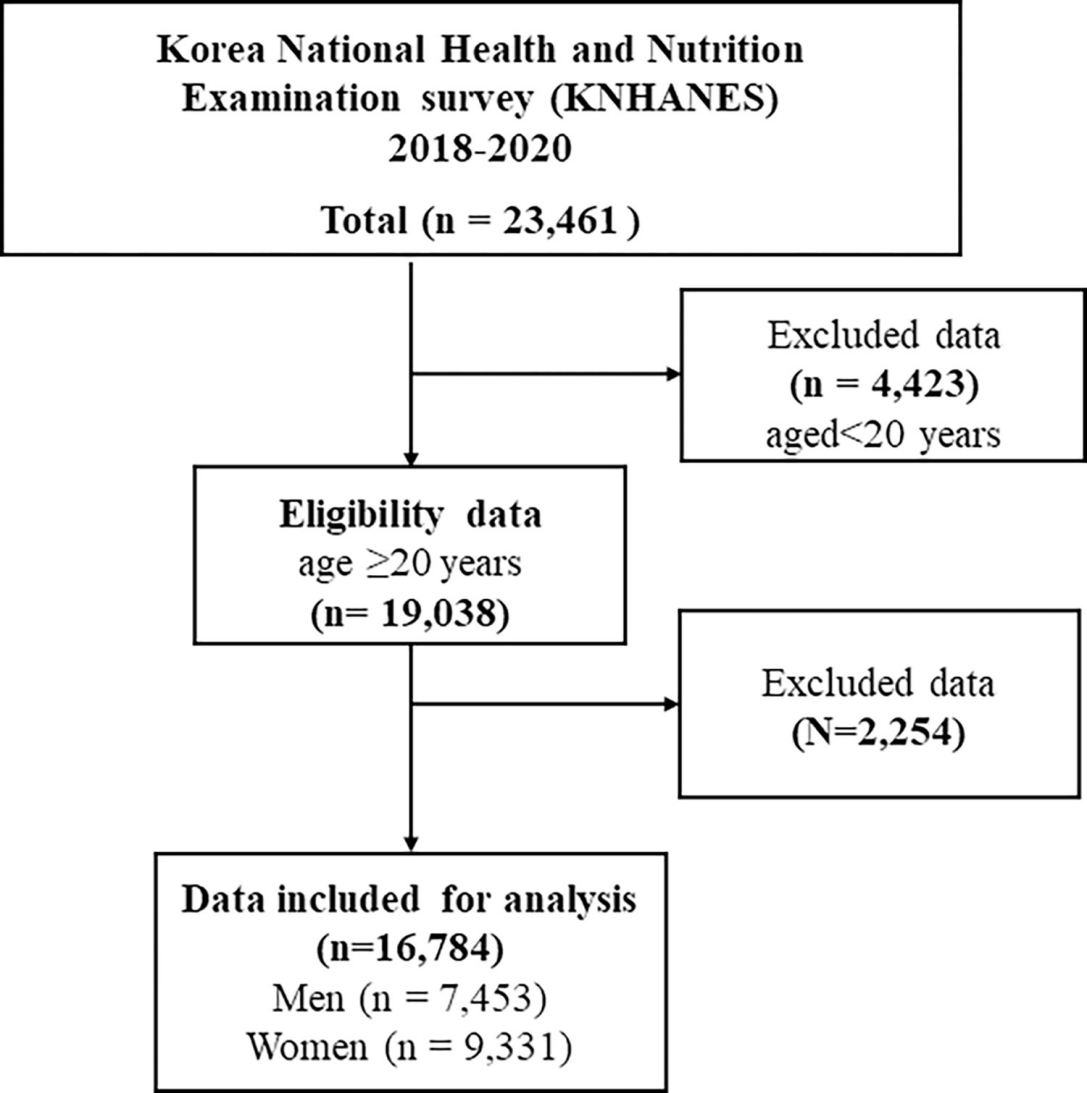
The flow chart of Population selection process (Korean National Health and Nutrition Examination Survey; 2018–2020).

The data were utilized from the KNHANES from 2018 to 2020, which is a standardized population-based survey and is based on a multi-stage clustered sample of the non-institutionalized South Korean population. The survey included a health interview, a nutritional survey, and a health examination survey. The survey collects information by field visit, through household interviews as well as direct, standardized physical examinations performed in specially equipped mobile examination facilities. The field survey team comprised of physicians, nurses and volunteers, they conducted face to face interview and physical examinations along with a mobile examination unit [24]. The primary sampling units (PSU) were selected, and the survey weights are provided by adjusting for complex survey designs with post-stratification. A total of 23,461 individuals are selected between 2018 and 2020. Of these, participants over 20 years (n = 19,038) are included in this study, and all the missed data of BMI, blood test data and obesity indicators are excluded. For the final analysis, 16784 participants were included in **Fig 1.**

The Institutional Review Board of the South Korean Centres for Disease Control and Prevention (approval no. 2013-07CON-03-4C) approved the KNHANES, conducted according to the Helsinki Declaration. The participants provided written informed consent to participate in this study.

### Variables

The dependent variable in this study was overweight and obesity, which were determined by BMI. BMI is calculated by dividing weight (kg) by the square of height (m^2^), as per the guidelines presented by the World Health Organization of the Asia Pacific Region and the South Korean Society for the Study of Obesity, which present the following definitions: underweight (< 18.5 kg/m^2^), healthy weight (18.5–22.9 kg/m^2^), overweight (23–24.9 kg/m^2^), and obese (≥25 kg/m^2^) [25].

The independent variables in this study were the individuals’ health behaviour pattern during the study period. The socio-demographic variables included age, sex, marital status, education, and household income. The age group was divided into five groups (20–29, 30–39, 40–49, 50–59, and over 60 years). The education status had four categories: elementary school, middle school, high school, and university and over. Marital status was classified as married and others (single, separated and divorced/widowed). The level of household income was calculated by dividing the household monthly net income and household size and classified into four categories: Q1 (lowest; <25th percentile), Q2 (Middle; 25th–49th percentile)), Q3 (high; 50th–74th percentile) and Q4 (Highest; ≥75th percentile) in the quartiles.

The lifestyle behaviours included smoking, alcohol consumption, self-perceived health status and body image, stress, and physical activity. Smoking was classified as a current smoker, a former smoker and never smoker. Drinking status was investigated using the Alcohol Use Disorder Identification Test (AUDIT),25 which consists of 10 questions related to alcohol consumption over the past year. Each item is scored from 0–4 points, giving a total range of 0–40 points. An AUDIT score ≤ 8 points is normal, between 8–15 points indicates alcohol abuse, 16–19 points indicates alcohol abuse requiring continued observation and ≥ 20 points indicates alcohol abuse requiring detailed examination.25 We have divided it into two groups: ‘No’ or ‘normal’(< 9 points) and ‘Yes’ or ‘abnormal’ (≥ 9 points or more). Self-reported health status was classified into three categories: fair (very good/good), moderate and bad (bad/very bad). In self-perceived stress level, the participants were asked for their perceived stress levels, and four options were then divided into two categories; the first two responses were considered ‘Yes’ (very much/much) and ‘No’ (Little/None). Self-reported body image in response to the question “What do you think of your current body image?” included normal (thin/normal) and obese (slightly obese/obese).

In terms of dietary habits, the frequency of breakfast, lunch and dinner and the use of dietary supplements were identified. The frequency of breakfast, lunch and dinner was considered from the response to “frequency of breakfast, lunch and dinner for one week during the past year” as ‘3 and more times (3–7 times/week) and less than 3 times (less than 3 times/week)’. The use of dietary supplements based on the response to ‘whether or not taking dietary supplements for more than 2 weeks in the past year’ was classified as ‘Yes’ or ‘No’. The metabolic variables were derived from the blood test values of blood pressure (systolic and diastolic pressure), fasting blood sugar level, triglyceride, and low HDL cholesterol. appear concurrently it was judged as metabolic syndrome.

### Statistical analysis

The KNHANES sampling weights accounting for the complex survey design, survey nonresponse and post-stratification were used. All the independent categorical variables were presented as frequency and percentage and compared with four categories of BMI by the chi-square test. Multivariable logistic regression was performed to measure the adjusted odds ratios (AORs) and 95% confidence intervals (CIs) for obesity and health behaviour pattern. All analyses were performed using SAS version 9.4.

MCA was performed to analyse the association between qualitative variables (sociodemographic, lifestyle behaviour, dietary habits, and metabolism) of an adults’ behavioural categories that were modified in to two groups to meet the MCA analysis and linked to variation in measures of BMI (overweight/obesity). The participants that remained from the initial population for the measures were considered. Two solutions were explored using the variable principal normalization method, although no defined number of dimensions is firmly established; some authors recommend a two-dimensional picture of data. Thus, MCA can suggest unexpected dimensions and relationships in the tradition of exploratory data analysis by applying to a Burt matrix or an indicator matrix [26]. The Burt matrix is a symmetric matrix of all two-way cross-tabulations between the categorical variables, and the indicator matrix is a matrix with cases as rows and categories of variables as columns. All statistical analysis and plots were performed in SPSS version 26.0.

## Results

### Socio-demographic characteristics

**Fig 2:**
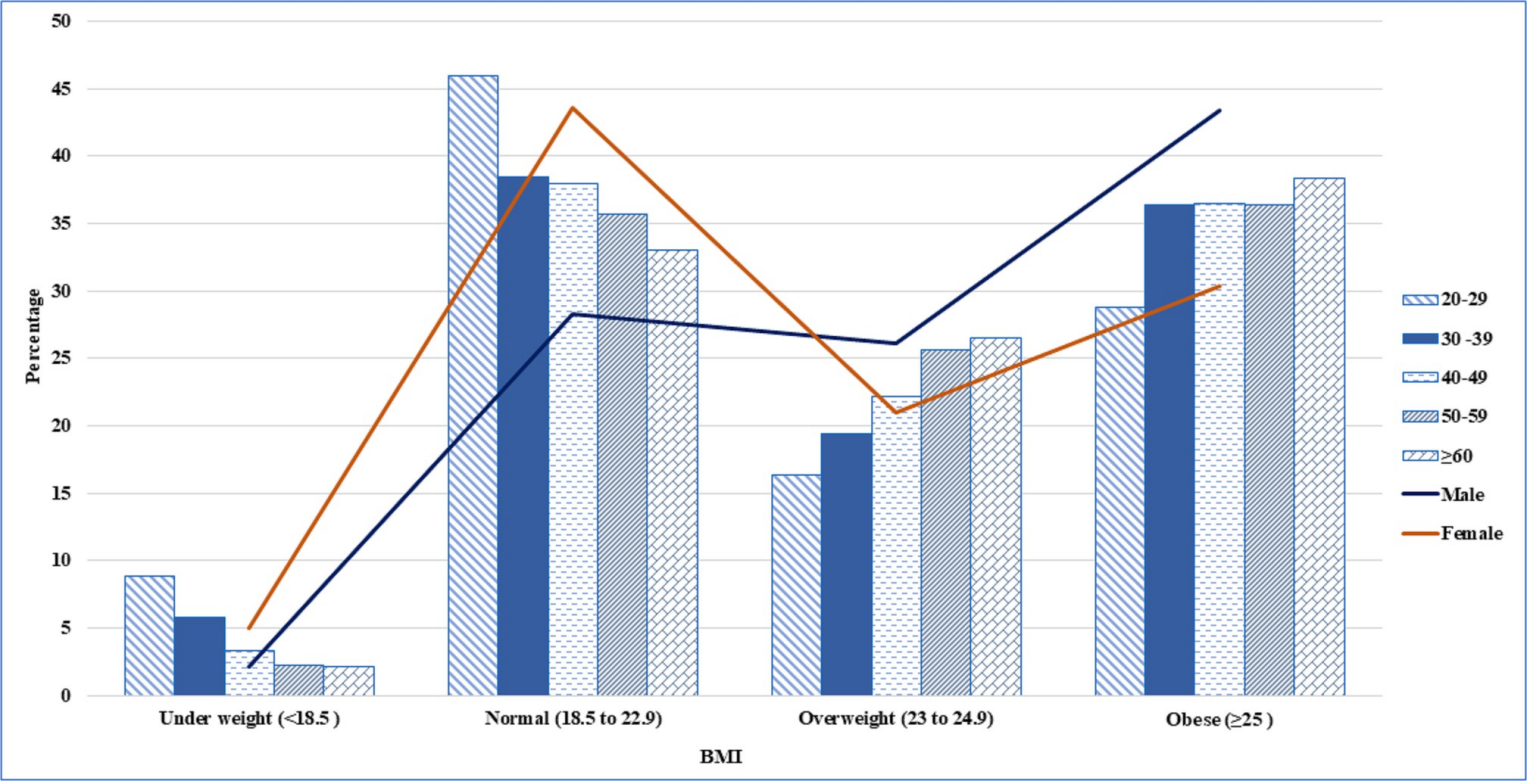
The percentage distribution of BMI according to the age groups and sex (Male and female).

There were 16,784 adults, including 7,453 men (44.4%) and 9,331 women (55.6%) given in Fig 1. Sociodemographic, health habits, dietary habits and metabolic factors are presented in **Table 1.** Of these BMI categories were underweight (3.8%), normal (36.8%), overweight (23.3%) and obese (36. 2%). According to the age group, most of them were over 60 years (38.3%), men (43.4%) and married (37%), as well as with an elementary level of education (40.7%) and an income level of Q1 and Q2. Percentage distribution by the age group and sex are showed in Fig 2.

**Table 1:** Baseline characteristics of South Korean adults according to health pattern behavior and BMI.

Variables		BMI category, kg/m^2^										
		Total		Under weight (<18.5)		Normal (18.5 to 22.9)		Overweight (23 to 24.9)		Obese (≥25)		
		N	%	N	%	N	%	N	%	N		%
		16,784	100.0	632	3.8	6,178	36.8	3,905	23.3	6,069		36.2
**Sociodemographic characteristics**												
Age (Years)	20–29	2,049	12.2	183	8.9	940	45.9	336	16.4	590		28.8
	30–39	2,448	14.6	142	5.8	941	38.4	475	19.4	890		36.4
	40–49	3,036	18.1	100	3.3	1,154	38.0	675	22.2	1,107		36.5
	50–59	3,231	19.3	73	2.3	1,154	35.7	827	25.6	1,177		36.4
	≥60	6,020	35.9	134	2.2	1,989	33.0	1,592	26.5	2,305		38.3
Sex	Male	7,453	44.4	166	2.2	2,112	28.3	1,944	26.1	3,231		43.4
	Female	9,331	55.6	466	5.0	4,066	43.6	1,961	21.0	2,838		30.4
Marital status	Married	13,774	82.1	406	3.0	4,922	35.7	3,355	24.4	5,091		37.0
	Others	3,010	17.9	226	7.5	1,256	41.7	550	18.3	978		32.5
Education	Elementary	3,088	18.1	101	2.2	981	32.3	750	24.7	1,252		40.7
	Middle school	1,642	9.8	41	2.5	543	32.0	444	26.1	648		39.5
	High school	5,596	33.5	204	3.7	2,082	37.2	1,307	23.4	2,003		35.8
	College and over	6,458	38.6	286	4.9	2,572	39.8	1,404	21.7	2,166		33.5
Income	Q1	4,160	24.8	232	4.3	1,494	36.4	898	21.9	1,536		37.4
	Q2	4,189	25.0	142	3.4	1,480	35.3	996	23.8	1,571		37.5
	Q3	4,222	25.2	165	3.9	1,545	36.6	984	23.3	1,528		36.2
	Q4	4,213	25.2	148	3.5	1,641	39.0	1,011	24.0	1,413		33.5
**Lifestyle characteristics**												
Smoking	Yes (Present)	3,831	22.8	81	2.1	1,131	29.5	1,016	26.5	1,603		41.8
	former	10,002	59.6	443	4.4	4,074	40.7	2,196	22.0	3,289		41.8
	never	2,951	17.6	108	3.7	972	33.0	693	23.5	1,176		39.9
Alcohol consumption	Yes	15,008	89.4	573	3.8	5,533	36.9	3,486	23.2	5,416		36.1
	No	1,776	10.6	61	3.3	644	36.3	419	23.6	652		36.8
Perceived Health status	Good	13,654	81.4	511	3.7	5,146	37.7	3,282	24.0	4,715		34.6
	Not good	3,130	18.6	121	3.9	1,032	33.0	623	19.8	1,354		43.3
Stress	Yes	4,462	26.6	198	4.4	1,660	37.2	920	20.6	1,684		37.8
	No	12,322	73.4	436	3.5	4,517	36.7	2,985	24.2	4,384		35.6
Self-perceived Body image	Underweight	2,468	14.7	507	20.5	1,735	70.4	165	6.7	61		2.5
	Normal	6,706	40.0	124	1.9	3,671	54.7	2,043	30.5	868		12.9
	Obese	7,610	45.4	3	0.0	771	10.1	1,697	22.3	5,139		67.5
Physical activity	Low	12,973	77.3	525	4.0	4,700	37.4	3,112	23.2	4,636		35.4
	Moderate	3,626	21.6	107	3.0	1,267	34.9	850	26.4	1,402		35.7
	High	185	1.1	34	18.4	68	36.8	45	24.3	38		20.5
**Metabolic characteristics**												
Waist circumference	Underweight	9,276	55.3	631	6.8	5,713	61.6	2,214	23.9	718		7.7
	Normal	3,368	20.1	210	6.2	612	18.2	1,173	34.8	1,373		40.8
	Obese	4,140	24.7	102	2.5	440	10.6	1,520	36.7	2,078		50.2
Hypertension	Yes	5,101	30.4	300	5.9	500	9.8	1,220	23.9	3,081		60.4
	No	11,683	69.6	332	2.8	5,678	48.6	2,685	23.0	2,988		25.6
Diabetes	Yes	2,484	14.8	10	0.4	412	16.6	413	16.6	1,649		66.4
	No	14,300	85.2	622	4.3	5,766	40.3	3,492	24.4	4,420		30.9
Cholesterol	High	1,700	10.1	23	1.4	528	31.1	431	25.4	718		42.2
	Low	15,084	89.9	609	4.0	5,650	37.5	3,474	23.0	5,351		35.5
TG	High	1,370	8.2	10	0.7	313	22.8	296	21.6	751		54.8
	Low	15,414	91.8	622	4.0	5,865	38.0	3,609	23.4	5,318		34.5
HDL	High	5,774	34.4	28	0.5	1,713	29.7	1,989	34.4	2,044		35.4
	Low	11,010	65.6	604	5.5	2,765	25.1	3,631	33.0	4,010		36.4
**Dietary habits**												
Breakfast (Times/Week)	≥ 3 times	9,194	58.2	481	5.2	3567	38.8	2201	23.9	2945		32.0
	≤ 2 times	6,590	41.8	151	2.3	2,611	39.6	1,704	25.9	2,124		32.2
Lunch (Times/Week)	≥ 3 times	10,811	75.8	520	4.8	3869	35.8	3551	32.8	2871		26.6
	≤ 2 times	5,973	24.2	112	1.9	2309	38.7	354	5.9	3198		53.5
Dinner (Times/Week)	≥ 3 times	10,952	79.1	479	4.4	3602	32.9	2578	23.5	4293		39.2
	≤ 2 times	5,832	20.9	153	2.6	2576	44.2	1327	22.8	1776		30.5
Supplement diet intake	Yes	7,335	46.8	325	4.4	2858	39.0	2094	28.5	2058		28.1
	No	8,449	53.2	307	3.6	3320	39.3	811	9.6	4011		47.5

### Multiple correspondence analysis

**Fig 3:**
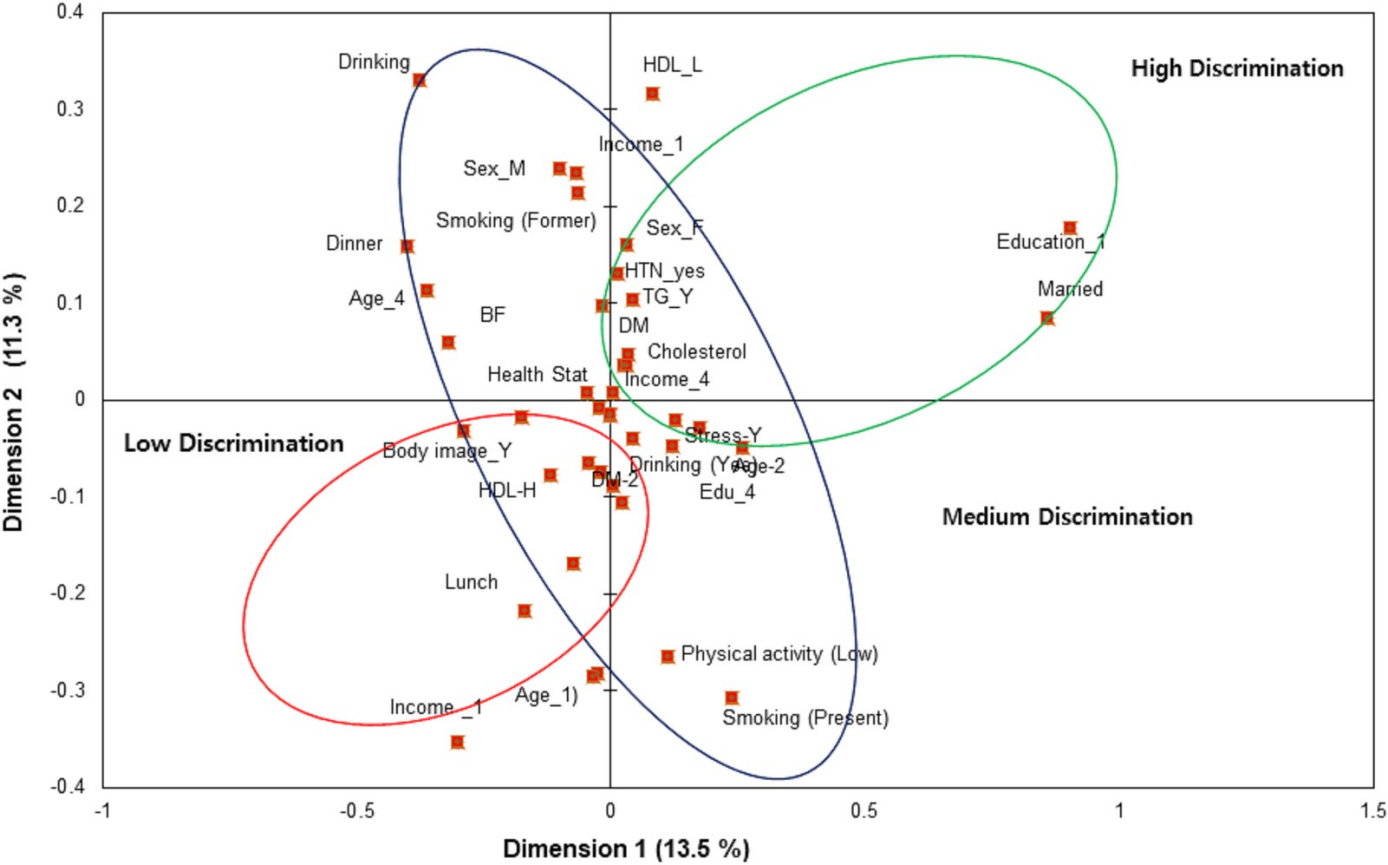
MCA dimensions; MCA dimensions discrimination measures by Joint category plot of the BMI categories (Overweight/Obesity) categories. HTN = Hypertension; DM = Diabetes; HDL = High-density lipoprotein; BF = Breakfast.

MCA analysis results are presented in **Table 2**; a two-dimensional MCA was considered the most adequate. The dimensions first and second presented an eigenvalue of 2.848 and 2.369 and inertia of 0.136 and 0.113, respectively. Discrimination measures and a joint plot of category points were presented in **Fig 3,** differentiating values were allocated to each of the obtained dimensions; all discrimination mean measures were below 0.5 with a maximum value of 0.464 (age-over 60 years) for the first dimension and 0.399 (sex-female) for the second dimension. The most discriminant variables for dimension 1 hierarchically were age, income, diabetes, physical activity, education, and cholesterol. In dimension 2, the most discriminant variables were sex, cholesterol, and blood glucose.

**Table 2:** MCA dimensions discrimination measures for overweight /obesity and covariates.

Covariates	Categories	MCA Dimension		Mean
		1	2	
Sex	Male/Female	0.017	0.399	0.208
Age (years)	≤ 59 / ≥60	0.464	0.187	0.275
Income	Low_1 /high_4	0.323	0.001	0.162
Education	School level_1 /College & over_4	0.240	0.093	0.167
Marriage	Married/others	0.199	0.077	0.138
Smoking	Former/present	0.024	0.168	0.096
Drinking	Yes/No	0.088	0.193	0.141
Stress	Yes/No	0.014	0.018	0.016
Health Status	Good/Bad	0.096	0.008	0.052
Body image	Good/Bad	0.047	0.141	0.094
Physical activity	(High/Low)	0.268	0.004	0.036
Hypertension	Yes/No	0.176	0.138	0.157
Diabetes	Yes/No	0.308	0.027	0.167
Cholesterol	Yes/No	0.242	0.325	0.284
TG	Yes/No	0.110	0.131	0.120
Blood Glucose	Yes/No	0.100	0.210	0.155
HDL	Low/High	0.161	0.108	0.134
Breakfast	Yes/No	0.152	0.105	0.129
Lunch	Yes/No	0.007	0.007	0.007
Dinner	Yes/No	0.007	0.005	0.006
Supplement diet	Yes/No	0.005	0.024	0.015
**Active Total**		2.848	2.369	2.558
**% Of variance**		0.136	0.113	0.122

TG = Triglycerides, HDL = High-density lipoprotein.

### Association between health pattern behaviour and overweight/obesity in South Korean adults

**Table 3**. presents the ORs with 95% confidence intervals of the multivariable logistic regression; covariates that were significantly associated with overweight and obesity were found. After controlling the subcategories, age (over 60 years), income (high), smoking, low physical activity, diabetes, high blood cholesterol and high triglycerides were significantly associated with a BMI linked to overweight and obesity (*P*<0.001). The ORs and 95% CIs for overweight and obesity across the four categories of BMI are presented in **Table 3.**

**Table 3:** Multivariable logistic regression analysis for association between overweight and obesity and health pattern behavior.

		BMI category, kg/m2											
Co-Variates	Under weight	Normal				Overweight (23 to 24.9)				Obese (≥25)			
	Ref	OR	95%CI		*p*	OR	95%CI		*p*	OR	95%CI		*p*
Sex (Male)	1.00	0.26	0.20	0.34	< .001*	0.06	0.05	0.08	< .001*	0.03	0.02	0.04	< .001*
Age (≥60 years)	1.00	1.28	1.15	1.42	< .001*	1.59	1.41	1.78	< .001*	1.53	1.35	1.72	< .001*
Income (Q4)	1.00	1.13	1.04	1.23	0.005*	1.26	1.15	1.39	< .001*	1.21	1.10	1.34	< .001*
Education (Low)	1.00	0.89	0.79	1.00	0.048*	0.81	0.71	0.92	0.005*	0.66	0.58	0.75	< .001*
Marital status (Yes)	1.00	0.78	0.59	1.03	0.079	0.90	0.64	1.26	0.534	0.92	0.65	1.29	0.615
Smoking (Present)	1.00	1.13	0.98	1.31	0.096	1.21	1.02	1.43	0.029	1.29	1.08	1.53	0.004
Alcohol intake (Yes)	1.00	0.88	0.63	1.23	0.465	0.74	0.51	1.09	0.128	0.61	0.41	0.90	0.012*
Stress (Yes)	1.00	0.99	0.80	1.21	0.893	1.14	0.90	1.44	0.286	1.08	0.85	1.38	0.538
PA (Low)	1.00	1.50	0.67	3.34	0.321	1.42	1.59	3.42	0.038*	3.23	1.79	4.69	0.021*
Diabetes (yes)	1.00	3.17	1.54	6.51	0.002*	4.57	2.16	9.65	< .001	12.91	6.11	27.29	< .001*
Blood Glucose (High)	1.00	1.00	0.48	2.08	0.990	0.74	0.34	1.61	0.452	0.72	0.33	1.56	0.401
Cholesterol (High)	1.00	2.08	1.30	3.34	0.002*	2.58	1.56	4.26	< .001*	2.70	1.62	4.50	< .001*
TG (High)	1.00	0.65	0.53	0.79	< .001*	0.49	0.39	0.62	< .001*	0.37	0.29	0.47	< .001*
HDL (Low)	1.00	2.00	1.36	2.94	< .001*	2.82	1.88	4.21	< .001*	3.98	2.65	5.97	< .001*

* p-value <0.05 significance level; PA = Physical activity, TG = Triglycerides, HDL = High-density lipoprotein.

## Discussion

This study aimed to find the association between health pattern behaviour and overweight and obesity by using data from the KHNANES during 2018–2020 and analysed the MCA-based discrimination by categorical data of sociodemographic, lifestyle behaviour, diet habits and metabolic variables by two-dimension variability. The study findings suggest that overweight and obesity are strongly associated with older age, and women had a high prevalence of obesity. This is consistent with the previous findings [15,16,19,20].

Our findings revealed that as age increases, the proportion of overweight and obesity increases. This contrasts with the previous report that a higher proportion of obesity was found in the age group between 25–55 years of early and middle-aged adults [8], and also, low level of education, high income, low physical activity, metabolic conditions of cholesterol, glucose and diabetes [10–13]. Other epidemiologic studies reported that smoking [27], low dietary pattern [12], alcohol consumption [10], and psychological distress [11,12] are associated with obesity. Additionally, the overweight and obese showed favourable discrimination with diet habits and lifestyle behaviour such as smoking and stress [14,19,20,27,28] at medium level. It was also previously reported that education inequality [11], socioeconomic factors [12–17] and history of smoking were more likely in overweight than underweight participants [3,8,10]. Moreover, stress was higher in the healthy weight group than in the underweight group, but similar in the overweight and obesity groups.

Furthermore, we found that low physical activity was highly associated with obesity. This is consistent with the reduction in physical activity observed in obese individuals [8,14,17,27,28]. Continuation of improvement in monitoring the regular physical activity would help to guide development of health policies and intervention programs to increase the physical activity levels especially for working group, thereby reducing the overall burden of obesity related chronic disease. Collaboration with community agencies would play a crucial role in successfully implementing such plans. For example, making use of existing transportation systems may assist people in attending health screenings and visiting local gyms and organizations might be offered related to wellness activities to reduce the obesity chances.

The MCA findings exhibited high discrimination of metabolic conditions of diabetes, high cholesterol, glucose, and low HDL and sociodemographic characteristics aged over 60 years, women, married and lower education level had high discrimination in MCA graphics. These results agreed with reports that obese groups have higher blood lipids, hypertension and diabetes, and that those who scored well in self-perception about obesity made greater efforts to improve their lifestyle [12]. Glycated haemoglobin, systolic blood pressure and total cholesterol levels in the overweight and obesity groups were significantly higher than healthy weight group. This corroborates evidence reporting that obesity is significantly associated with hypertension, diabetes, and hyperlipidaemia [4,8]. Additionally, our results indicate medium discrimination approaches in the irregular diet habits such as breakfast and dinner and lifestyle behaviour such as stress, smoking and body image. In this regard, improper diet and healthy lifestyle strategies are less likely to promote overweight and obesity. On the other hand, government public health proposals to improve the awareness of healthy lifestyle and diet practices by initiating nutrition education programmes may have greater success in overcoming the obesity rate among adults, especially in the working age group.

The multivariable logistic analysis revealed that overweight and obesity according to the BMI categories were better among individuals in young adult groups and having higher education qualifications than high and middle school education. This finding would be attributed to the higher likelihood of an individual with lower education having lifestyle and diet habits with regular physical activity. The present study provides meaningful findings that modifiable factors such as lifestyle and diet habits should be improved to prevent obesity prevalence. It was observed that characteristics of individuals’ socioeconomic status, diet habits and biological activity accounted for an important component of the geographical distribution of overweight [2,3,5,6,10,13,15,18,20]. Understanding the socioeconomic status differences and relevant aspects of built environments can inform the development of programs aimed at reducing obesity [23,28,29]. The present findings simply imply that increase the clear evidence in a global context in public health concern to be current body of knowledge.

However, there were some limitations in our study. First, we present study employed a cross-sectional data. Therefore, it could not be confirming the causal association and limits the interpretation of the exact results. Second, we did not analyse the comorbidity that could associated with the overweight and obesity. Third, information on quality of diet habits is not added due to lack of data, such as breakfast and energy intake and reasons for skipping diet habits are unknown. Also, we have included one question of only self-perceived stress in health status. However, some studies have been reported, depression or other related variables are highly associated with the obesity and eating behaver by sensitivity specificity and negative prediction [30]. Nevertheless, this study has the advantages of using the population-based representative data that provides a health behaviour pattern related to obesity and overweight and real data from blood tests rather than using self-reported data. Fourth, To the best knowledge, this is the first study investigated the MCA based differences in the association between health behaviour pattern and overweight and obesity. However, use of MCA found some illustrative purposes, it could be liable, in case of three or more categorical variables will be considered, and continuous variables may not be appropriate depending on the context. MCA yields principal inertias (eigenvalues) that considerably underestimate the quality of the results in a low-dimensional space. Traditionally, MCA involves transferring bustle table in to two dimensions by mean in simple form. Although such transformation does not always preserve some of the true multivariable association. Since the data only coded by the categories and each individual behaviour belongs, an alternative measure of fit to count how well these categories are predicted by the solution. A wide range of exposure variables was selected to establish the MCA although continues variables could not include in this study, especially in consideration of some physiological or biological factors, which have been suggested to have an association with obesity.

## Conclusion

This study examined the association between individual health behaviour pattern and overweight and obesity for South Korean adults aged 20 years or older. The present study findings showed that as age increases, men rather than women showed a tendency to overweight or obesity. It was found that lower education level, current smoking and drinking, low physical activity, increased glycaemia, high total cholesterol, low HDL, and hypertension were associated, and the score also increased statistically significantly. Unhealthy food habits and low physical activity are likely to endure, increasing the risk for adults for obesity and chronic diseases in the future, unless public health initiatives are designed to be multilevel and take into consideration the multiple correlates that influence the behaviour surrounding diet intake and physical activity. Further research on health behaviour patterns should focus on linking identified factors to important clinical outcomes to identify vulnerable groups and to allow for patient-centred primary prevention programs and interventions to make efforts with innovative programs. Additionally, self-care management programs related to dietary habits may help to prevent primary diseases such as diabetes, hypertension, and hyperlipidaemia and secondary diseases such as stroke and cardiovascular disease. Moreover, they may also help to reduce medical costs both nationally and individually and increase the subjective quality of life. This study suggests, future studies should investigate the association between diet consumption or energy intake and overweight obesity such as quantity, quality, and frequency.

## Abbreviations

KNHANES: Korean National Health and Nutritional Examination Survey
MCA: multiple correspondence analysis
BMI: body mass index
OR: odds ratio
CI: confidence interval
WHO: World Health Organization
KSSO: Korean Society for the Study of Obesity
PSU: primary sampling units
AUDIT: Alcohol Use Disorder Identification Test
HDL: high-density lipoprotein
HTN: Hypertension
DM: Diabetes
BF: Breakfast
